# Response surface optimization for xylanase with high volumetric productivity by indigenous alkali tolerant *Aspergillus candidus* under submerged cultivation

**DOI:** 10.1007/s13205-012-0077-1

**Published:** 2012-07-26

**Authors:** Debabrata Garai, Vineet Kumar

**Affiliations:** Biochemical Engineering Laboratory, Department of Chemical Engineering, Indian Institute of Technology, Roorkee, Uttarakhand India

**Keywords:** *Aspergillus candidus*, Box-Behnken design, Submerged fermentation, Xylanase, Volumetric productivity

## Abstract

In this study, a novel isolate *Aspergillus candidus* was employed for xylanase production using low cost agro residues. A Box-Behnken design matrix was used to optimize the influential parameters like carbon source, nitrogen source and incubation temperature for maximum xylanase production. Under optimized condition, enzyme titer level enhanced to 69 IU/ml at 48 h with volumetric productivity 1437 IU/l h. Growth and enzyme production were observed even at pH 11.0, indicating its ability to sustain at high alkaline environment. Little amount of cellulase was produced concomitantly with xylanase during the course of the process. Volumetric productivity of xylanase was found as a function of temperature. This fungal strain was emerged as a one among few strains having high xylanase productivity.

## Introduction

Xylanases are group of enzymes mainly consisting of endoxylanase (EC 3.2.1.8) which primarily cleaves β-1, 4 linked xylan backbone and β-xylosidase (EC 3.2.1.37) which converts xylooligomers to monomeric xylose sub unit (Biely 1985). Xylanases are produced by bacteria, yeast and filamentous fungi. Among the mesophilic fungi, the species of *Aspergillus*, *Penicillium*, and *Trichoderma* are well-known xylanase producers (Bakri et al. 2003; Li et al. 2007a; Shah and Datta 2005; Silva and Carmona 2008). Microbial xylanases have attracted the attention of many researchers primarily because of their application in various industries like functional food additives (Aachary and Prapulla 2009), improvement of bread quality (Jiang et al. 2008), biofuel (Tabke et al. 2006), and paper and pulp industries (Taneja et al. 2002).

Cost of raw material has direct impact on overall production of enzymes. Industrial use of pure xylan is prohibitive for large scale production of xylanase due to its high cost. Since India is an agro-based country, it generates huge quantity of agro waste. Use of several agro-industrial residues like wheat bran, wheat straw, corn cob, etc. has been explored to make the process cost effective (Li et al. 2007a; De Souza et al. 1999; Katapodis et al. 2007).

Optimization of process development using ‘classical one factor at a time’ is time consuming and does not consider interaction effect among variables. On the other hand, statistical design offers an alternative and efficient approach for process optimization. Response surface methodology has been widely used as a tool for optimization of different process parameters used in different process development. It can evaluate and predicts the interaction among critical variables.

Box-Behnken optimization, a class of rotatable second-order design based on three level partial factorial designs, was selected to study the effect of influential variables such as carbon source, nitrogen source and temperature on the response.

Volumetric productivity is a measure of effectiveness of the bioprocess development. Bioprocess with high productivity is always desirable for scale up of the process to industrial level as it minimizes operating time and thereby operating cost of the process. Many investigators reported agro industrial residues as a preferential substrate for effective xylanase production but strains with high productivity are few (Table 1).

**Table 1:** Comparison of volumetric productivity of xylanase of different fungal strain using agro residues as a substrate

Micro organism	Substrate	Xylanase Activity (IU/ml)	Xylanase productivity (IU/l h)	References
*Aspergillus flavus*	3 % Corn cob	190	660	De Souza et al. (1999)
*Pleurotus ostreatus*	2.5 % Corn cob + 2.5 % wheat bran	25	149	Qinnghe et al. (2004)
*Chaetomium thermophilum*	3.9 % Wheat straw	61	635	Katapodis et al. (2007)
*Penicillium oxalicum*	3.5 % Wheat bran	14.5	102	Li et al. (2007b)
*Aspergillus nidulans*	2 % Wheat bran	40	417	Taneja et al. (2002)
*Thermomyces lanuginosus*	3 % Corn cob	1,439	8,564	Gomes et al. (1993)
*Aspergillus niger*	3 % Rice straw + 1 % wheat bran	620	6,458	Min et al. (2007)
*Aspergillus foetidus* MTCC 4898	2 % Wheat straw	20	278	Shah and Datta (2005)
*Aspergillus fumigatus*	3 % Corn cob 3 % Wheat straw	125 119	1,302 1,240	Lenartovicz et al. (2002)
*Aspergillus ochraceus*	0.6 % Wheat bran 0.6 % Rice straw	45 37	268 220	Biswas et al. (1990)
*Trichoderma viride*	2 % Wheat straw	72	500	Gomes et al. (1992)
*Fusarium oxysporum* F3	2 % Corn cob	245	2,552	Christakopoulos et al. (1996)
*Penicillium janthinellum* NCIM 1171	1 % Bagasse	130	902	Adsul et al. (2004)
*Emericella nidulans* KK-62	2 % Wheat bran	362	2,155	Kango et al. (2003)
*Aspergillus candidus*	1.17 % Wheat bran	69	1,437	This work

To the best of our knowledge, it was the first report of xylanase production by alkali tolerant *Aspergillus candidus.* In the present study, production and optimization of xylanase with high productivity using inexpensive agro residues have been described.

## Materials and methods

### Microorganism

*Aspergillus candidus* was isolated from a soil sample collected from Meerut Institute of Engineering and Technology (MIET) campus, Meerut, Uttar Pradesh, India by dilution plate method. The pure culture obtained by single spore isolation was grown and maintained on 2 % malt extract agar. These pure culture slants were stored under refrigerated conditions. The strain has been identified and deposited at Agharkar Research Institute, Pune, India (NFCCI 2314) and Institute of Microbial Technology (IMTECH), Chandigarh, India, bearing the accession no. MTCC 10743.

### Xylanase production

An Erlenmeyer flask (250 ml) containing 50 ml of modified basal liquid medium (Nakamura et al. 1993) containing (g/l) oat spelt xylan 5.0; peptone 5.0; yeast extract 5.0; potassium dihydrogen phosphate (KH_2_PO_4_) 1.0; and magnesium sulfate (MgSO_4_·7H_2_O) 0.1 was sterilized at 121 °C for 15 min and then cooled to room temperature. The initial pH of the medium was adjusted to 6.2 and was not further controlled. The flask was inoculated with four inoculum discs (10 mm diameter) of the culture grown for 7 days on PDA medium. Each disc contained approximately 6 × 10^4^ spores. The inoculated flasks were incubated at 25 °C for 5 days on a rotary shaker with 150 rpm. At the end of the incubation period, the contents of each flask were pooled and filtered through Whatman no. 1 filter paper. The filtrate containing the crude enzyme was used in further analysis.

### Biomass determination

The mycelia mass present on pre-weighed filter paper was washed several times with distilled water to remove soluble substances and dried in oven at 70 °C until a constant weight was obtained.

### Protein determination

Protein concentrations in cell free supernatant were measured by the Lowry method (Lowry et al. 1951) with bovine serum albumin as a standard.

### Enzyme assay

Xylanase activity was quantitatively assayed in cell free supernatant according to the method of Bailey et al. (1992). The reaction mixture (2.0 ml) containing 1.0 ml of 1.0 % (w/v) oat spelt xylan in 0.1 M citrate buffer, pH 5.0; 0.9 ml citrate buffer and 0.1 ml of a suitably diluted enzyme solution was incubated at 55 °C for 5 min. The reaction was stopped by adding 3 ml of 1.0 % dinitro salicylic acid reagent. The reaction mixture was then kept on boiling water bath for 10 min. Amount of reducing sugars liberated was determined by measuring absorbance of the resulting color at 540 nm in a UV–vis spectrophotometer using xylose as standard. (Miller 1959).

One unit (IU) of xylanase activity was defined as the amount of enzyme that produced 1 μmol of xylose equivalent per min of reaction and per ml of enzyme solution under the assay condition.

CMcase activity was determined by similar method using 1 % (w/v) carboxy methyl cellulose as substrate and d-glucose as standard. Controls without either enzyme or substrate were run simultaneously. Each experiment was carried out in triplicate and results were taken as the mean of three values.

### Kinetics of xylanase production using different pure sugar and complex agro residues

Xylanase enzyme production was monitored by growing *A. candidus* on the basal medium containing oat spelt xylan 5.0 g/l as a sole source of carbon. The inoculated flask was incubated for 5 days and samples were harvested at every 24-h intervals.

Xylan was replaced by different low cost agro residues such as wheat bran, rice bran, wheat straw, sugar cane bagasse and corn cob and pure sugars such as glucose, maltose, lactose, xylose, starch, CM-cellulose, fructose, sucrose in the basal media at a concentration of 5.0 g/l. Wheat straw, sugar cane bagasse, corn cob were hammered in a ball mill and sieved through 0.710 mm screen size before use. All the inoculated flasks were incubated at 25 °C for 48 h. Content of each flask was filtered through Whatman no. 1 filter paper and filtrates were used for xylanase, CMCase and extracellular protein assay.

### Effect of different nitrogen sources on xylanase production

To assess the effect of various organic and inorganic nitrogen sources on enzyme production, mixture of yeast extract and peptone in the basal medium was replaced with other organic nitrogen sources, such as peptone, yeast extract, beef extract, soybean meal and inorganic nitrogen sources urea, ammonium sulfate ((NH_4_)_2_SO_4_), ammonium nitrate (NH_4_NO_3_), sodium nitrate (NaNO_3_), di-ammonium hydrogen orthophosphate ((NH_4_)_2_HPO_4_), which were individually added to the basal medium at a concentration of 10.0 g/l. Wheat bran was used as a carbon source. All the inoculated flasks were incubated at 25 °C for 48 h.

### Effect of pH on xylanase production

To evaluate the effect of initial culture pH of the basal liquid media on xylanase production, the initial pH of the media was adjusted to a value between 2.0 and 12.0 before sterilization using 1 M HCl and 1 M NaOH. Wheat bran at a concentration of 5.0 g/l was used as carbon source. After 48 h, enzyme activities were determined in each flask as described before.

### Effect of temperature on xylanase productivity

To study the effect of temperature on the production of xylanase, the inoculated flasks were incubated at different temperatures (25, 30, 35, 40 and 45 °C) for 5 days with constant shaking at 150 rpm. Oat spelt xylan was replaced by wheat bran in the production medium. pH of the media was kept at 6.0. Samples were withdrawn at every 24-h intervals and enzyme activities were determined as before.

### Optimization of different variables for xylanase production: response surface methodology

A statistical method, Box-Behnken design was adopted to optimize most important variables to maximize xylanase production. The carbon source, wheat bran (*A*), nitrogen source, mixture of peptone and yeast extract in equal concentration (*B*) and temperature (*C*) were studied as the independent variables to maximize response. The experimental design consisted of 17 experiments of three variables (*A*, *B*, *C*) at three levels (+1, 0, −1). The independent variables were coded −1 and +1 as low and high levels. The experimental range, level and code of independent variables are shown in Table 2. All the experiments were done in triplicate and average xylanase activity obtained was taken as dependent variable or response (*Y*).

**Table 2:** Experimental range, level and code of independent variables

Independent variables	Symbol coded	Range and levels		
		−1	0	+1
Wheat bran (g/l)	*A*	2	10	18
Peptone and yeast extract (g/l)	*B*	2	10	18
Temperature (°C)	*C*	20	25	30

The following second-order polynomial equation was adopted to study the effects of variables to the response.1where *Y* is the response (xylanase activity), β_0_ is the constant term; β_1,_ β_2_ and β_3_ are the coefficient of linear terms; β_11,_ β_22_ and β_33_ are the coefficient of quadratic terms; and β_12,_ β_13_ and β_23_ are the coefficient of cross product terms, respectively.

The goodness of fit of the polynomial equation was expressed by coefficient of determination *R*^2^ and its statistical significance level was checked by *F* test. The quality of fit also verified by calculating absolute average deviation (AAD) defined as2where *y*_*i*,exp_ and *y*_*i*,pre_ are the experimental and predicted responses, respectively, and *p* is the number of experimental run. Design-Expert (version 6.0.8) Stat-Ease, Inc., soft ware was used for regression analysis and graphical representation of the data obtained. The independent variables were optimized using desirability function criteria available in Design-Expert software. The aim was to maximize xylanase activity while keeping the variables in their respective experimental range. Using these criteria, desirability was kept maximum, i.e., 1.0. The algorithm of the desirability criteria used was as follows.

Experiments were carried out in triplicate using the optimized condition to validate the result from the analysis of the response surface. Experimental results were statistically attested by calculating standard error prediction (SEP).where *y*_*i*,e_ is experimental value of the *i*th experiment, *y*_p_ is the model predicted xylanase activity and *y*_e_ is the average value of xylanase activity of three experimental run and *n* is the number of experiment conducted.

### Scale up of xylanase production in 5 L bioreactor

Bioreactor (Biotron, LiFlus GX) with working volume of 5.0 L, equipped with two six-blade turbine impellers was used to scale up xylanase production. Nutritional components were kept identical to that of shake flask culture. Fungal spores scraped from the top surface of freshly prepared malt extract agar media and transferred into 200 ml sterile distilled water containing approximately 5 × 10^4^ spores/ml were used as inoculums. Initial pH of the sterile culture medium was kept at 6.0. Agitator speed and aeration rate were fixed at 150 rpm and 0.5 vvm, respectively. Fermentation was allowed to continue for 120 h at 25 °C and samples were taken at regular interval for enzyme assay.

## Results and discussion

### Kinetics of xylanase production using different pure sugar and complex agro residues

**Fig. 1:**
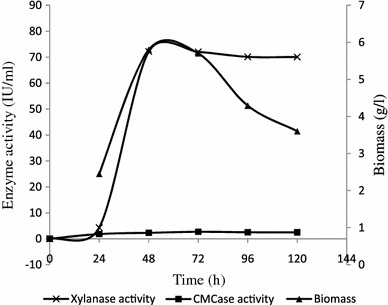
Kinetics of xylanase, cellulase and biomass production using xylan as a substrate

The time course of xylanase production using xylan as a carbon source was monitored by measurement of xylanase activity, CMCase activity and dry biomass (Fig. 1). Xylanase production was found to commence at 24 h and reached a maximum (72.16 IU/ml) at 48 h of incubation. Then, it was remained almost constant up to 72 h and slowly decreased thereafter. Cell growth was started at 24 h and reached maximum (5.8 g/l) at 48 h. It suggested that cell growth and enzyme were synthesized simultaneously. Low amount of carboxymethyl cellulase was concomitantly produced during the course of the reaction and maximum cellulase activity was found 2.72 IU/ml at 72 h. Volumetric productivity of xylanase (a measure of effectiveness of the bioprocess) was found to be maximum (1,503.34 IU/l h) at 48 h. Medium pH was raised around 8.0 within 24 h and slowly increased to 8.48 at 120 h. It suggests that *A. candidus* can survive at alkaline pH and showed natural tolerance of enzyme towards alkaline environment. Rise of medium pH may be associated with release of nitrogenous protein metabolites in the media (Silva et al. 2005). Similar observation was also found in xylanase production by *Aspergillus fumigatus* (Anthony et al. 2003).

**Fig. 2:**
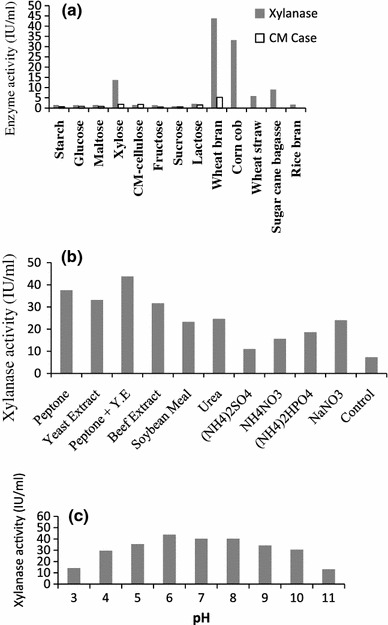
Effect of (**a**) carbon sources; (**b**) nitrogen sources and (**c**) pH on enzyme production. The shake flask experiments were performed for 48 h at 25 °C, 150 rpm. Control refers production media without any external nitrogen source

Natural substrates such as wheat bran, wheat straw, rice bran, sugarcane bagasse, corn cob and pure sugars such as glucose, maltose, xylose, starch, CM-cellulose, fructose, sucrose and lactose were exploited to replace high cost pure oat spelt xylan and thereby making the process cost effective. Their effect on enzyme production has been shown in Fig. 2a. Among the natural carbon substances, wheat bran was found to be best inducer of xylanase production (43.82 IU/ml) followed by corncob (33.20 IU/ml). The production of xylanase using sugar cane bagasse and wheat straw was found extremely low and rice bran was found to be an ineffective inducer for xylanase production. CMCase synthesis was low in the presence of corn cob (1.45 IU/ml) compared to wheat bran (5.20 IU/ml). Although higher xylanase synthesis (72.16 IU/ml) was observed when pure oat spelt xylan was used as a substrate, it was replaced by wheat bran as it is cheap and easily available material producing best enzyme yield and hence preferentially employed in all further experiments. Superiority of pure xylan substrate over agro residues on enzyme synthesis has also been reported in *Trichodermainhamatum* (Silva and Carmona 2008), *Thermomyces lanuginosus* (Damaso et al. 2000), and alkali tolerant *Aspergillus niveus* (Sudan and Bajaj 2007). On the contrary, wheat bran has been found as a best inducer for xylanase production in *Penicillium oxalicum* (Li et al. 2007b)*, Aspergillus nidulans* (Taneja et al. 2002). These suggest that affinity to substrates for xylanase synthesis is depending on the nature of strain. Among the pure sugar tested, xylose was found to support enzyme synthesis at a certain level (13.71 IU/ml), but much less compare to wheat bran and corn cob. All the pure sugar tested was found to be an excellent growth promoter, but ineffective inducer for xylanase synthesis. Similar observations have also been reported in xylanase production by other fungi. (Silva and Carmona 2008; Gomes et al. 1994). Finding of this study was in accord with other researchers where complex carbohydrates reported as a preferential source for xylanase biosynthesis, however, pure sugars such as xylose and lactose were found to induce xylanase production in certain fungal strain (Anthony et al. 2003; Damaso et al. 2000; Royer and Nakas 1989; Xiong et al. 2004).

### Effect of nitrogen sources on xylanase production

Xylanase production was studied in the presence of different organic and inorganic nitrogen sources using wheat bran (5 g/l) as a substrate given in Fig. 2b. Organic nitrogen sources like peptone and yeast extract when used in equal quantities supported better synthesis of the enzyme than any of the organic and inorganic nitrogen sources used alone. Similar result was also reported in xylanase production in solid state fermentation by *Penicillium canescens* where combination of yeast extract and peptone gave the best result (Bakri et al. 2003). Preferential use of organic nitrogen sources in the synthesis of xylanase has widely demonstrated by earlier investigators (Sudan and Bajaj 2007; Qinnghe et al. 2004). Enzyme titer was found extremely low (7.26 IU/ml) in the absence of any nitrogen sources, indicating nitrogen content in wheat bran was not sufficient to support higher level of enzyme synthesis. Among the inorganic nitrogen sources, sodium nitrate was found as a best source of nitrogen although not comparable to organic nitrogen. Although ammonium sulfate was known to promote xylanase synthesis in certain fungal strain found unsuitable in the present investigation (Seyis and Aksoz 2005).

### Effect of pH on xylanase production

pH of the production media was adjusted from 2.0 to 12.0 to evaluate its effect on xylanase biosynthesis (Fig. 2c). The culture has wide range of pH tolerance growing from pH 3.0 to 11.0. Slightly acidic or near neutral pH like 6.0 was found to be the best for the maximum synthesis of xylanase (43.82 IU/ml) and little cellulase (5.208 IU/ml). Considerable amount of xylanase enzyme titer was observed at alkaline pH, indicating alkali tolerant nature of the fungus. Present organism of our study is one among the few alkali-tolerant *Aspergillus* strain found in the literature. Other alkali tolerant fungi of the same genus producing xylanase are *Aspergillus flavus* (De Souza et al. 1999), *A. niveus* (Sudan and Bajaj 2007), *A. nidulans* (Taneja et al. 2002), *Aspergillus fischeri* (Senthilkumar et al. 2005) and *A. fumigatus* (Anthony et al. 2003).

### Effect of temperature on enzyme productivity

Submerged fermentation was carried out using wheat bran as a substrate at 25–40 °C for 120 h to evaluate its effect on growth and enzyme synthesis (Table 3). Maximum enzyme titer (43.82 IU/ml) was observed at 25 °C for 48-h incubation period and remained almost constant up to 72 h. Slight decrease in enzyme activity was observed at the end of 120 h. Further increase in incubation temperature to 40 °C inhibited the enzyme synthesis drastically. No growth was observed above 40 °C, suggesting mesophilic nature of the fungus. Increase in incubation temperature from 25 to 30 °C increased the enzyme synthesis nearly sixfold at 24-h incubation time. At 25 °C for 24 h incubation time, volumetric productivity (IU/l h) of xylanase was calculated as 206.25 IU/l h, increased to 1,217.08 IU/l h when temperature was increased to 30 °C. Even we consider volumetric productivity as an important parameter to assess the efficiency of the bioprocess, incubation at 30 °C was found ideal for maximum enzyme productivity. Nevertheless, lower incubation temperature like 25 °C was suitable for maximum xylanase production.

**Table 3:** Effect of incubation temperature on xylanase yield and productivity

Temperature (°C)	Xylanase yield (IU/ml)				Productivity of xylanase (IU/l h)			
	25	30	35	40	25	30	35	40
Time (h)								
24	4.950	29.21	27.66	2.913	206.25	1217.08	1152.50	121.37
48	43.82	34.92	28.32	4.814	912.91	727.50	590.00	100.29
72	43.50	33.16	25.96	1.903	604.16	460.55	360.55	26.43
96	42.92	32.19	23.50	1.783	447.08	335.31	244.79	18.57
120	39.80	31.85	19.50	1.100	331.66	265.41	162.50	9.168

### Optimization of independent variables: response surface methodology

To optimize carbon (wheat bran), nitrogen (mixture of yeast extract and peptone) sources and temperature, a Box-Behnken design, consisting of a set of 17 experiments with five replicates at central point was conducted. The Box-Behnken design matrix of the independent variables in coded units along with predicted and experimental values of response is given in Table 4. All the experiments were performed in 250 ml Erlenmeyer flask containing 50 ml of media. The quadratic model expressed by equation (3) represents xylanase activity (*Y*) as a function of wheat bran (*A*), mixture of peptone and yeast extract (*B*) and temperature (*C*).

**Table 4:** Box-Behnken design matrix along with the experimental and predicted values of xylanase activity

Run no.	*A*: Wheat bran (g/l)	*B*: Yeast extract + peptone (g/l)	*C*: Temperature (°C)	Xylanase activity (IU/ml)	
				Predicted	Observed
1	0	0	0	63.38	64.44
2	−1	0	−1	11.11	10.83
3	−1	0	+1	25.41	27.04
4	+1	−1	0	41.81	43.61
5	−1	+1	0	22.85	21.05
6	+1	+1	0	30.09	29.64
7	0	−1	+1	32.22	30.14
8	0	+1	−1	12.52	14.60
9	0	+1	+1	41.76	41.93
10	0	0	0	63.38	64.08
11	0	−1	−1	33.17	33.01
12	+1	0	−1	24.67	23.04
13	−1	−1	0	22.23	22.68
14	0	0	0	63.38	64.03
15	0	0	0	63.38	61.60
16	+1	0	+1	38.67	38.95
17	0	0	0	63.38	62.73

3

**Fig. 3:**
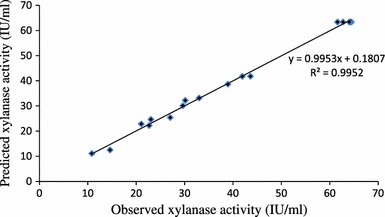
Parity plot showing distribution of experimental and predicted values of xylanase production

**Fig. 4:**
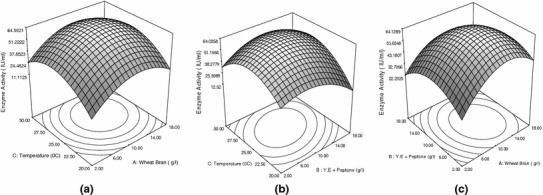
Response surface plot showing relative effect of two variables **a** wheat bran and incubation temperature; **b** temperature and mixture of peptone and yeast extract; **c** wheat bran and mixture of peptone and yeast extract

The statistical significance of the polynomial equation was checked by *F* test and analysis of variance (ANOVA) for response surface quadratic model is given in Table 5. The *p* value serves as a tool for checking the significance of each of the coefficient. Model terms having *p* value <0.05 were considered significant whereas less than 0.0001 treated as highly significant. Model *F* value was calculated as 161.80 and *p* value <0.0001 suggests the model is highly significant. It could be concluded from the Table 5 that coefficient of linear and quadratic effect of each model term wheat bran (*A*), mixture of peptone and yeast extract (*B*) and temperature (*C*) are significant. It is implying that both carbon and nitrogen source can act as limiting substrates and little change in their concentration will affect enzyme synthesis level (Tanyildizi et al. 2005). The *p* value of coefficient of model term *AB* and *BC* was 0.0159 and 0.0001, respectively, indicating significant interaction effect between two variables *A* and *B* as well as between *B* and *C*. The goodness of fit of the model was checked by calculating the coefficient of determination *R*^2^, adjusted *R*^2^ and analysis of AAD. The value of *R*^2^ is always in between 0.0 and 1.0. *R*^2^ value close to 1.0 implies that the model is accurate and predicts better response. However, model with higher *R*^2^ value always does not mean that model is accurate. Large *R*^2^ value also is resulted by addition of non-significant extra variables in the model. Thus, it may be possible of a model having higher *R*^2^ value with poor prediction of response. So the term adjusted *R*^2^ has been introduced which arranges the *R*^2^ values for the sample size and for the number of variables in the model. Addition of insignificant model term in the model leads to decrease in adjusted *R*^2^ value. So, the value of *R*^2^ should be as close as to adjusted *R*^2^ (Haaland 1989). Bas and Boyaci (2007) suggested performing the analysis of AAD as a method to check the accuracy of the model. Larger *R*^2^ value and smaller AAD value together can predict true behavior of the system more accurately. The *R*^2^ value was found to be 0.9952; indicating 99.52 % of the variability of the response could be explained by this model. Adjusted *R*^2^ value 0.9891 was very close to *R*^2^ value. Low AAD value (3.66 %) verified the model equation to be adequate to describe the experimental design. Adequate precision measures signal to noise ratio, a ratio greater than 4.0 is desirable. An adequate precision of 34.90 for xylanase indicated adequate signal. Non significant lack of fit (*p* value 0.0772) is good and indicates that the model equation was adequate for xylanase activity under any sets of combination of the variables. Coefficient of variation (CV) was found to be 5.08. Lower CV value indicated the experiments conducted were precise and reliable (Box et al. 1978). Parity plot (Fig. 3) showed the distribution of experimental and model predicted values where data points are localized close to the diagonal line suggesting accuracy of the model. The 3D plot (Fig. 4a–c) and their respective 2D contour plot provided a visual interpretation of the interaction between two factors. From 3D response plot, it can be concluded that model encompassed the optimum region for xylanase production at the peak of the surface and enzyme production decreased with extreme values of the variables. Figure 4a represents response surface plot between two variables wheat bran concentration (*A*) and temperature (*C*) where concentration of third variable (*C*) was kept at their respective zero level. It can be seen that maximum xylanase production obtained at intermediate concentration of wheat bran at near to room temperature. High concentration of wheat bran induced highly viscous media as fermentation continued and raised difficulty in proper agitation. This might be the cause of getting reduced enzyme activity at very high substrate concentration. Figure 4b represents response surface plot of two independent variables mixture of yeast extract and peptone (*B*) and temperature (*C*). It can be inferred that maximum xylanase synthesis took place when concentration of nitrogen source was kept at their middle level. Figure 4c represents response surface plot between wheat bran (*A*) and nitrogen source (*B*). It can be seen that maximum xylanase activity obtained when values of carbon and nitrogen sources were kept at their respective middle level. Interaction between variables can also be predicted by visualizing their respective contour plot. Elliptical nature of contour indicates significant interaction between the variables where as circular nature predicts insignificant interaction between variables. Point of intersection between major and minor axes gave the value of maximum point (Muralidhar et al. 2001). From the Fig. 4b, c, it could be concluded that there was a significant interaction effect between nitrogen source (*B*) and temperature (*C*); wheat bran (*A*) and nitrogen source (*B*) as it was evident from their respective *p* value also, whereas circular nature of contour (Fig. 4a) between wheat bran (*A*) and temperature (*C*) indicated insignificant interaction between them. The model predicted the optimum concentrations of *A*, *B* and *C* were 11.75 g/l, 9.98 g/l and 26 ± 0.25 °C, respectively, and maximum response 64.54 IU/ml.

**Table 5:** Analysis of variance (ANOVA) for the fitted quadratic model of xylanase activity as a function of independent variables

Source	SS	DF	MS	*F* Value	*p* > *F*
Model	5551.51	9	616.83	161.80	<0.0001^a^
*A*	359.66	1	359.66	94.34	<0.0001^a^
*B*	61.72	1	61.72	16.19	0.0050^b^
*C*	400.16	1	400.16	104.96	<0.0001^a^
*A* ^2^	1,608.12	1	1,608.12	421.82	<0.0001^a^
*B* ^2^	896.04	1	896.04	235.04	<0.0001^a^
*C* ^2^	1,498.95	1	1,498.95	393.18	<0.0001^a^
*AB*	38.07	1	38.07	9.99	0.0159^b^
*AC*	0.023	1	0.023	5.902E−003	0.9409^c^
*BC*	228.01	1	228.01	59.81	0.0001^a^
Lack of fit	21.06	3	7.02	4.99	0.0772^c^
Residual	26.69	7	3.81		
Pure error	5.63	4	1.41		

*R*^2^: 0.9952, adj *R*^2^: 0.9891, pre *R*^2^: 0.9380

C.V: 5.08, adequate precision: 34.90

*SS* sum of squares of model parameters, *DF* degree of freedom, *MS* mean square of model parameters

^a^Highly significant, *p* ≤ 0.0001

^b^Significant, *p* ≤ 0.05

^c^Non significant, *p* > 0.05

### Experimental validation of the optimized condition

Experiment was conducted with optimized conditions predicted by RSM analysis to verify the accuracy of the model. The xylanase activity was found to be 69 IU/ml after 48 h of cultivation. Experimental result was very close to the value predicted (64.54 IU/ml) by the model with standard error prediction (SEP) 3.73 %. CMCase activity in the enzyme solution was found to be 5.82 IU/ml.

Response surface optimization of the independent variables led to increase xylanase synthesis by 57.46 % compared to the xylanase production obtained by modified media composition (xylan was replaced by wheat bran) provided by Nakamura et al. 1993.

### Scale up xylanase production in 5 L bioreactor

**Fig. 5:**
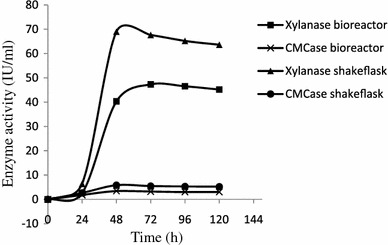
Comparison of xylanase and cellulase production in shake flask and bioreactor under optimized condition

Xylanase production profile in a batch fermenter using optimized culture medium has been studied (Fig. 5). At 24-h fermentation, very little amount of xylanase was produced (2.62 IU/ml). Enzyme production was found to increase gradually up to 47.33 IU/ml at the end of 60 h and remained almost constant up to 72 h. The xylanase activity slowly decreased thereafter and at the end of 120 h it was found 45.20 IU/ml. CMCase was produced with very low level and maximum activity observed at the end of 48 h (3.44 IU/ml). Media pH was found to rise with in 24 h cultivation and continued to increase slowly to a final value of 8.30 at the end of 120 h. The yield of xylanase in bioreactor (47.33 IU/ml) was less in comparison to that achieved in parallel shake flask culture (69 IU/ml). The cause of decreased enzyme titer level may be due to following factors.

- The suspended insoluble wheat bran particles were moved to upward direction under the influence of agitator speed leading to accumulation at the wall of the head space of the fermenter.
- Accumulated wheat bran particles at the liquid–air interface acted as a support to grow fungus more at top surface compare to bulk liquid.
- Thick mycelium mat was developed at the top of the vessel within 24 h of cultivation and clogged air outlet resulted difficulty in constant air supply.

Decreased enzyme synthesis in bioreactor in comparison to shake flask has been documented in other fungal xylanase also (Kumar et al. 2009; Gomes et al. 1994).

## Conclusions

In this work, *A. candidus* was identified as an important strain to produce xylanase with high volumetric productivity. This strain was found as a one among few fungal strains having higher xylanase productivity. Considering this aspect, the present organism is ideally suitable for commercial exploitation as it uses easily available low cost wheat bran as a substrate. It can sustain even in the alkaline environment to produce enzyme. Therefore, the problem of contamination generally remains minimal.
